# First-Principles Estimation of Core Level Shifts for Hf, Ta, W, and Re

**DOI:** 10.1021/acs.jpcc.2c00981

**Published:** 2022-05-24

**Authors:** Daniel Wolverson, Benjamin Smith, Enrico Da Como, Charles Sayers, Gary Wan, Luca Pasquali, Mattia Cattelan

**Affiliations:** † Centre for Nanoscience and Nanotechnology and Department of Physics, University of Bath, Bath BA2 7AY, United Kingdom; ‡ Centre for Nano Science and Quantum Information, University of Bristol, Tyndall Avenue, Bristol BS8 1FD, United Kingdom; § Department of Engineering, University of Modena and Reggio Emilia, Via Vivarelli 10, Modena 41125, Italy; ∥ School of Chemistry, University of Bristol, Cantocks Close, Bristol BS8 1TS, United Kingdom

## Abstract

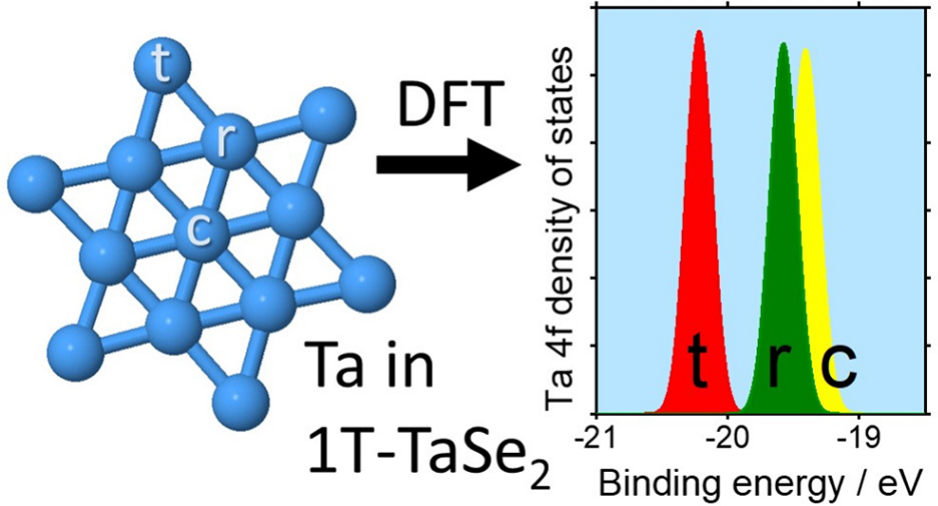

A simple first-principles approach is used to estimate the core level shifts observed in X-ray photoelectron spectroscopy for the 4f electrons of Hf, Ta, W, and Re; these elements were selected because their 4f levels are relatively close to the Fermi energy. The approach is first tested by modeling the surface core level shifts of low-index surfaces of the four elemental metals, followed by its application to the well-studied material TaSe_2_ in the commensurate charge density wave (CDW) phase, where agreement with experimental data is found to be good, showing that this approach can yield insights into modifications of the CDW. Finally, unterminated surface core level shifts in the hypothetical MXene Ta_3_C_2_ are modeled, and the potential of XPS for the investigation of the surface termination of MXenes is demonstrated.

## Introduction

X-ray photoelectron spectroscopy (XPS) is a powerful tool for the chemical analysis of near-surface atoms in the solid state and uses the fact that the binding energies of the core electron states are only weakly affected by the environment of a given atom (many textbooks discuss the definition of binding energy; for example, see Huefner^1^), leading to the ability to identify the presence of a chemical element by its XPS spectrum. However, the small shifts in the energy of a given core level are frequently measurable and can be diagnostic of the chemical environment of the atom.^2,3^ Nowadays, XPS is considered a mature characterization technique: this is due to (i) the availability of a huge database of XPS spectra in the literature for all of the elements in the periodic table in different environments and compounds, (ii) the availability of lab equipment with bright and focused photon sources and advanced electron analyzers, allowing for bidimensional real-space or *k*-space mapping with submicrometer lateral resolution,^4,5^ (iii) the exploitation of the photon tunability and high-energy resolution achievable at synchrotron light sources which couple a high brightness with a continuous range of excitation energies. A free choice of excitation energy also means that the investigator has some control over the core level photoabsorption cross section and over the depth below the surface over which the photoemitted electrons escape and can be detected.^1^

For many purposes, it is sufficient simply to identify a core level energy in order to recognize a particular element or, at the next level of detail, to apply an empirical calibration of the chemical shifts of the core level energy to identify different bonding environments. In addition, depending on the position of the photoemitting atom in the system under study, further effects can influence the binding energy: final state effects, such as differential core hole screening, or band bending. For instance, it is well known how to recognize the oxidation states of many metal species or to distinguish oxides from the native state of a given element. The core level shifts (CLS) in question are typically on the order of tens to hundreds of millielectronvolts and thus represent a small fraction of the binding energies, which are several tens of electronvolts. This means that with a typical energy resolution in XPS of ∼100 meV, fitting of the XPS spectra is required to extract the peak positions and compare to tabulated values, but this fitting is not challenging unless there are closely similar and overlapping peaks to be taken into account. Lineshape details are complicated, but standard approaches have emerged and are well established^6^ with excellent guidance being available for nonexperts.^7−9^

Despite the usefulness of empirical approaches, much effort over several decades has been devoted to the fundamental problem of predicting the shifts in core levels due to the chemical environment. In principle, this requires consideration of (i) the ground state of the electronic system before excitation, (ii) the response of the system to the positive charge left by the photoemitted electron, and (iii) the vacuum state of the photoemitted electron after emission. This implies that a model is required for both the bulk ground and excited states and possibly the surface state of the material.^10,11^

Here, we consider a simple approach to modeling the ground state core level shifts in the particular case of the transition metal elements Hf, Ta, W, and Re. If its reliability can be established, such a simple approach is useful because it can easily be incorporated into models of more demanding situations (for example, to look at dynamical changes in the core level shifts when modeling time-resolved photoemission data^12−16^ or to study the complex behavior of adsorbates^17^). We test the approach using the surface core level shifts of each of these four metals before applying the same approach to the different Ta sites in the charge density wave (CDW) material 1T-TaSe_2_ and the hypothetical “MXene” Ta_3_C_2_ with comparisons to experimental XPS data where possible. In these cases, and many more cases of this type, the transition metals occupy more than one type of site within the lattice and the CLS between sites may be obtained within the framework of a single DFT calculation as follows.

## Methods

We used the ab initio density functional theory method (DFT) as implemented in the plane-wave code Quantum Espresso^18,19^ to approximate the ground state wave function via Kohn–Sham theory.^20^ We used either scalar or fully relativistic projector augmented wave (PAW) pseudopotentials^21^ from the PS library in which, crucially, the 4f orbitals were included as valence states, and we used the Perdew–Zunger exchange-correlation functional^22^ in the local density approximation (LDA). We confirmed that results were similar using the Perdew–Burke–Ernzerhof generalized gradient approximation (PBE-GGA) functional.^23^ The use of fully relativistic pseudopotentials introduces the spin–orbit splitting between th 4f_5/2_ and the 4f_7/2_ manifolds; in all cases, the core level shifts were found to be the same for both.

Apart from the first example below, the structures were relaxed with respect to the in-plane lattice parameters and atomic coordinates were freely optimized to obtain forces of less than 10^–3^ eV Å^–1^ per atom. Convergence with respect to the kinetic energy and charge density cutoffs was confirmed, and Monkhorst–Pack *k*-point grids^24^ of at least 8 × 8 × 1 were used for the metal slabs; for monolayer 1T-TaSe_2_ in the commensurate CDW (CCDW) phase with a  unit cell, a 6 × 6 × 1 grid was used. Adjacent layers were separated by a vacuum of 15 Å or more. Since the 4f_5/2_ and 4f_7/2_ manifolds show the same core level shifts, our calculations for CCDW material 1T-TaSe_2_ did not take spin–orbit splitting into account in order to reduce the computational time; the calculation requires over 500 valence electrons when the 14 4f electrons at each of the 13 Ta sites in the  CCDW unit cell are included.

As noted above, our approach uses two key simplifications. First, we neglect any final state effects (or, equivalently, we assume that final state effects affect the final state energies of all 4f photoelectrons equally), and we avoid the need for the construction of a pseudopotential to represent the core–hole state after electron emission. Tardio et al. described this approach as “very crude” but still “useful”.^25^ We should point out, however, that the final state contributions can sometimes be considerable, as has been illustrated for the example of the 4d electrons of Mo, Rh, Pd, and Ag, where decomposition of the initial and final state contributions to the total SCLS is given.^26^ It should be noted that more exact approaches using specially constructed pseudopotentials to model the core–hole final states are problematic for crystalline systems with periodic boundary conditions,^10^ since one is dealing with a charged state, while much of the recent effort has concentrated on representing the core–hole states of relatively light elements (C, N), so suitable core–hole pseudopotentials for the transition metals are not readily available but have to be generated from scratch and tested. The significant effort involved in this makes the investigation of a simpler approach worthwhile.

Second, we assume that the Kohn–Sham eigenvalue for the 4f states mimics the response of the real core levels to changes in the atomic environment. At the Hartree–Fock level of approximation, in which electron correlation effects are neglected, the identification of the initial state binding energy with the single electron core level eigenvalue is exact (this is Koopmans’ theorem^2,27^), and even this level of approximation can give a useful estimate of the experimental binding energy.^11^ However, Koopmans’ theorem does not hold for the Kohn–Sham eigenvalues of a DFT calculation; nevertheless, core level *shifts* can be predicted by Koopmans’ theorem applied to Kohn–Sham eigenvalues.^10,28^

The elements chosen here, Hf, Ta, W, and Re, are special because their most characteristic XPS peaks arise from the 4f level with particularly low binding energies (∼14.3, 21.7, 31.3, and 40.6 eV, respectively, for 4f_7/2_).^29^ Their 4f states thus lie relatively close in energy to the 5d valence electrons. In view of this, it is often desirable in pseudopotential-based calculations to include the electrons of the filled 4f^14^ orbital as valence (rather than core) electrons, though this implies an extra computational cost and is not always essential. When the 4f states are treated as valence states, solution of the Kohn–Sham Hamiltonian in DFT yields their associated energy eigenvalues. These can then be linked with specific lattice sites by calculating projections of the Kohn–Sham wave functions onto the atomic states localized at each site. If these sites differ in, for example, the local charge density, this gives rise to small shifts of the 4f energy eigenvalues. We used the postprocessing code projwfc within the Quantum Espresso suite to project the wave function onto the orthogonalized atomic wave functions using PAW projectors and PAW all-electron basis functions^30^ to obtain the partial density of 4f states for each metal site.

The XPS spectrum of bulk 1T-TaS_1.2_Se_0.8_ shown in Figure 3 was acquired at the Spectromicroscopy beamline of the Elettra synchrotron at 94 K, positioning the analyzer for normal emission from a submicrometer spot of a freshly cleaved sample. The photon energy was 74 eV, and the pass energy was set to 20 eV with an overall energy resolution of about 50 meV.

## Surface Core Level Shifts of Metal Slabs

To investigate whether these predicted Kohn–Sham core level shifts are a useful guide to the behavior of real systems, we first consider the surface core level shifts (SCLS) of slabs of the four transition metals. High-quality LEED data on their low-index metal surfaces are available, and since XPS is highly surface sensitive, reliable experimental measurements of the SCLSs and associated modeling are also available. The case of Ta was studied recently, and the general correspondence between the Kohn–Sham energies and the experimental binding energies was noted,^31^ but that work concentrated on the absolute 4f binding energy and did not investigate the shifts in binding energies at nonequivalent Ta sites.

**Figure 1:**
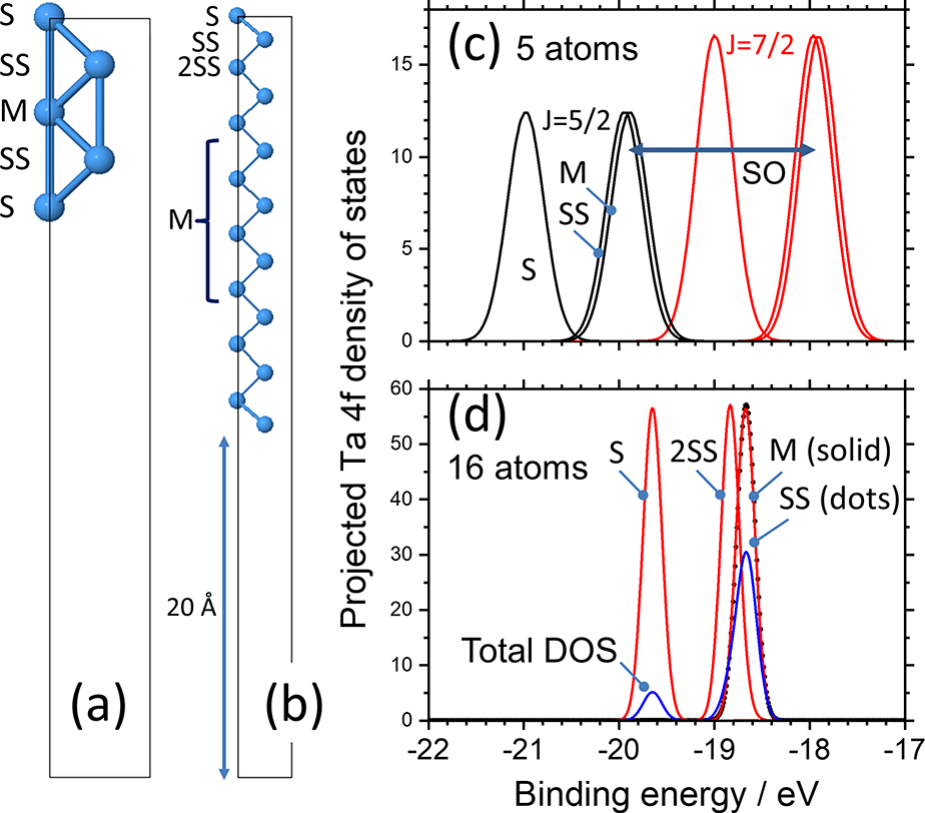
Surface core level shifts of the tantalum (001) surface. (a) Side view of an unrelaxed 5-layer slab, (b) side view of a relaxed 16-layer slab, and (c) predicted Ta 4f binding energies for the surface (S), subsurface (SS), and middle (M) layers of the unrelaxed 5-layer slab on the same energy scale as d. Horizontal arrow labeled SO indicates the magnitude of the spin–orbit splitting between the 4f_5/2_ and the 4f_7/2_ manifolds. (d) Predicted Ta 4f binding energies for the surface (S), middle (M), subsurface (SS), and second subsurface layer (2SS) in the relaxed 16-layer slab, calculated without spin–orbit interaction (SS peak, shown dotted, coincides with M). In blue, the total density of 4f states summed over all Ta sites is plotted, scaled for ease of comparison of the peak position.

Figure 1 shows results for the case of Ta, which is our main focus because of current interest in the two-dimensional CDW material 1T-TaSe_2_. We modeled two types of slabs having 5 and 16 layers of atoms in vacuum (Figure 1a and 1b, respectively) with the body-centered cubic (bcc) α-Ta structure and (001) surfaces. For 5 layers, Figure 1a, the Ta sites are of three types: surface (S), subsurface (SS), and middle (M). For 16 layers, Figure 1b, we find that the central 6 atoms of the slab behave as representative M sites with a calculated layer spacing of 1.64 Å, in good agreement with the experimental bulk layer spacing of 1.65 Å (which is one-half the cubic lattice parameter, *a* = 3.3058 Å).^32^

Our choice of 5 layers follows an earlier work, which found a shift between 4f core levels of the S and M sites of *E*_SM_ = 960 meV with no appreciable core level shift between M and SS sites.^33^ In that study, all atoms were located on their equilibrium experimental bulk lattice sites, that is, without relaxation. For consistency, we also used this unrelaxed configuration (but with the calculated bulk lattice parameter obtained with our choices of pseudopotential and exchange-correlation functional rather than an experimental lattice parameter), and our results are shown in Figure 1c. They agree with the previous linearized augmented plane wave (LAPW) calculations,^33^ giving a lattice parameter of *a* = 3.224 Å and SCLS of *E*_SM_ = 1045 meV. However, this latter result is a significant overestimate when compared to experimental SCLS values, which range from 750 ± 5^34^ and 740 ± 10 meV^35^ to 718 ± 5 meV.^36^ To address this, first, we increased the number of layers to 16 to improve the representation of the bulk (M) atoms, and second, we allowed all atomic coordinates and in-plane lattice constants to relax to minimize the forces on all atoms. Experimental evidence for the need to take the surface relaxation into account for Ta (001) was reported earlier,^32,34^ but there is a consensus that there is no symmetry-breaking reconstruction of this surface.^32−34^

We find a considerable relaxation of the surface layer of atoms, so that the bond length between atoms S and SS is not typical of the bulk (M) atoms, as indicated in Figure 1b. A 14% contraction of the surface interlayer spacing (that is, between the S and the SS planes) has been obtained from the analysis of angle-resolved photoemission and XPS measurements,^34^ while low-energy electron diffraction (LEED) and X-ray diffraction (XRD) studies gave an 11% contraction of the surface layer spacing and a 1% expansion of the subsurface layer spacing (between SS and 2SS).^32^ Our computational results are in broad agreement with this picture, predicting a 12% contraction of the surface layer spacing. However, we find a 1% *contraction* of the SS–2SS spacing and a 3% expansion of the layer spacing below (2SS–3SS), so our predicted surface relaxation appears more complex than those used to interpret experimental data. Our computational result is in agreement with a previous DFT calculation,^37^ which also found oscillatory changes in layer spacing, independent of the choice of pseudopotential.

The associated changes in charge density affect the 4f core level shifts of the S, SS, and 2SS atoms, resulting in the spectrum shown in Figure 1d. As in the case of the unrelaxed structure, the surface core level (S) shifts to larger binding energy than that of the bulk atoms (M), and there is also a significant shift between the 2SS and the M atoms though; surprisingly, the core level shift of the SS atoms is within 10 meV of that of M. These shifts correlate with the changes in the interlayer spacing discussed above. Bearing in mind that our predicted M and SS contributions are too close to resolve experimentally, we could approximate the experimental SCLS by the splitting between S and 2SS (because they are the two peaks most energetically distinct from the bulk). This gives an SCLS of 816 meV, in better agreement with the experimental values given above. For the relaxed 16-layer slab, the splitting of the surface state S from the mean of the subsurface layers is 930 meV, in poorer agreement but still an improvement on the unrelaxed case.

**Figure 2:**
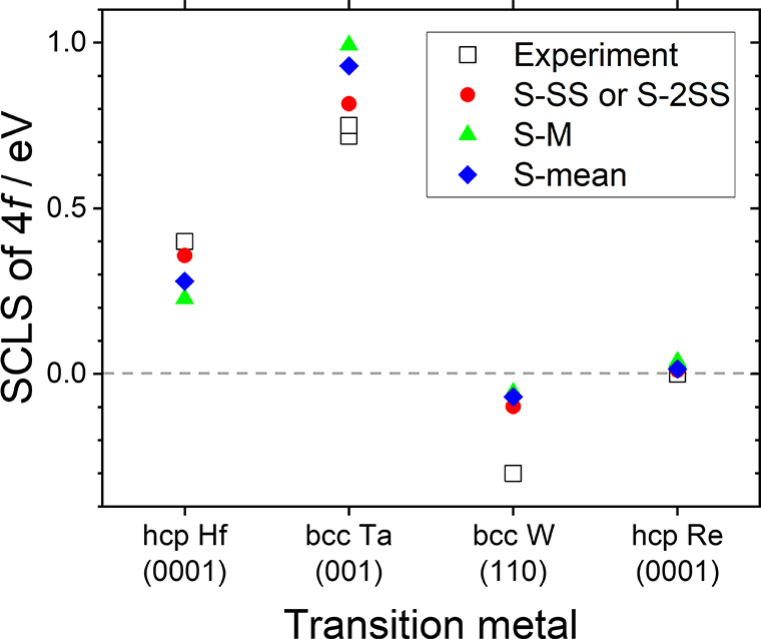
Surface (S) 4f core level shifts from the subsurface (SS) and central (M) atoms of relaxed 8-layer thick slabs of the four transition metals; surface orientations were (0001) for the hcp metals Hf and Re, (001) for Ta, and (110) for W: (black squares) experimental data summarized from sources discussed in the main text; (red circles) shift between S and SS (except in the case of Ta, where it is the shift between S and 2SS); (green triangles) shift between S and M; (blue diamonds) shift between S and the mean of SS and M.

The same procedure gives estimates of the SCLSs for all four transition metals, which are shown in Figure 2, which is based on Figure 4 of Mrårtensson et al.^38^ but compares predicted and experimental data for the (001) Ta surface^35^ rather than that of the (110) surface. For the case of W, however, we modeled the (110) surface since it is believed to be stable with respect to reconstruction.^39^ We show the predicted splittings of S from both SS and M and the mean of SS and M (and for Ta the splitting between S and 2SS). In each case, the agreement with the experimental results also shown in Figure 2 is reasonably good. The trends in the magnitude and sign of the SCLSs and the small or zero SCLSs in the cases of W and Re have been discussed elsewhere and are linked to the occupancy of the 5d level in these particular metal surfaces;^38^ note that the SCLSs of these elements are not necessarily small in other chemical contexts, as we shall see below in our discussion of the MXenes. It should be emphasized again that for each metal we are comparing the SCLSs of different atomic sites in the same input structure and obtained within the same DFT calculation; we are not making any assumption about the accuracy of the absolute binding energies of the 4f levels, and so we do not attempt to interpret, for example, the shift between the centroids of the 4f levels in Figure 1c and 1d. This very simple approach does however reproduce the trend of the experimental data and gives immediate insight into how the SCLS shifts depend on the surface relaxation.

## Core Level Shifts of Charge Density Wave Materials

**Figure 3:**
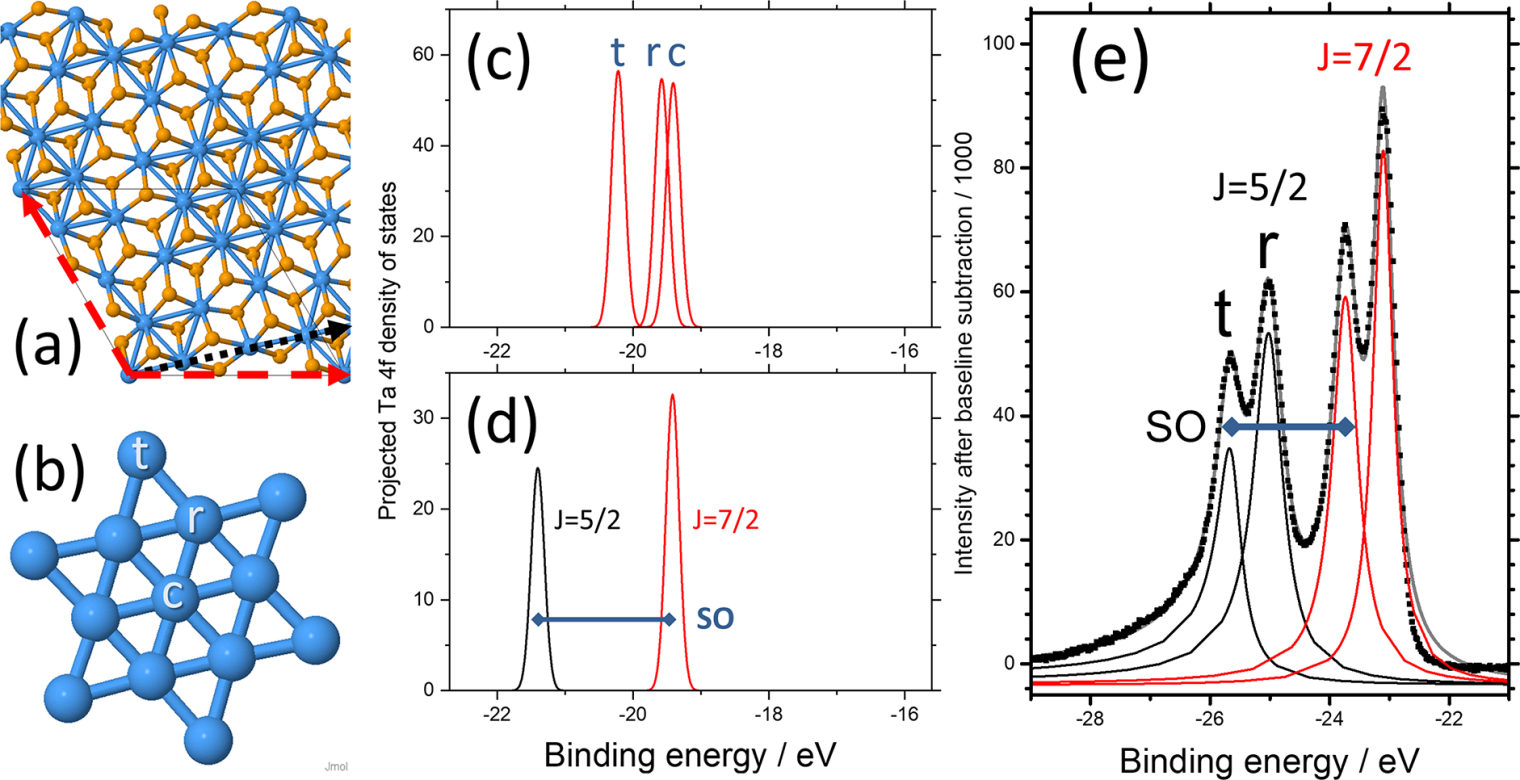
Commensurate charge density wave (CCDW) phase of 1T-TaSe_2_. (a) Top view of a single layer with Ta and Se atoms colored blue and yellow, respectively. Dotted black vector shows the *a* axis of the normal structure, and dashed red vectors show the axes of the CCDW phase. (b) View of the “Star of David” formed by the displacement of the Ta atoms in the CCDW phase with labels indicating the tip, ring, and central atoms (t, r, and c, respectively). (c) Predicted binding energies of the Ta 4f XPS signals from single t, r, and c atoms (without spin–orbit splitting). (d) Predicted XPS signals for the normal phase of 1T-TaSe_2_, including spin–orbit splitting SO. (e) Experimental XPS spectra (dots) of CCDW 1T-TaS_1.2_Se_0.8_ showing fitted contributions from t and r atoms (black and red lines for *J* = 5/2 and 7/2 components, respectively, and gray line for the overall fit).

Good reviews of charge density waves in materials of the transition metal dichalcogenide family are available elsewhere;^40−42^ here, we will focus directly on 1T-TaSe_2_. This material, as mentioned above, shows a commensurate charge density wave (CCDW) phase below 473 K with a  reconstruction in the layer plane, as shown in Figure 3a, giving a “Star of David” structure in which there are three nonequivalent Ta sites (the tips, inner rings, and center positions of the stars, shown in Figure 3b and labeled t, r, and c, respectively).^43^ In this structure, the lattice remains hexagonal but the in-plane crystal axes are rotated in plane by 13.89° with respect to the unreconstructed lattice, as shown by the black dotted and red dashed vectors in Figure 3a. The modulation of the charge density due to the CCDW gives significantly different charge densities at the three sites (t, r, and c) and lifts their crystallographic equivalence; this also leads to significant core level shifts between their 4f levels. The usefulness of the 4f CLS as a probe of the amplitude of the charge density wave was recognized very early in the study of these materials,^44−46^ and simple models were developed to interpret them, based on the superposition of cosinusoidal plane waves having the CDW wavevector and symmetry.^45^ These models are still employed in investigating modifications to the basic commensurate phase.^47^ However, the response of the 4f CLS is also ideally suited to modeling via the present methodology, since all three Ta sites are modeled within a single DFT calculation, the essential criterion for this approach.

For simplicity in this initial study, we have not included on-site electron–electron interactions via, for example, a DFT+U approach. Our level of approximation is known to reproduce (for example) the instability of the unreconstructed phase with respect to the CCDW phase,^48,49^ the temperature dependence of this instability, the frequencies of the lattice modes, and the pressure dependence of the phase transition.^50^ However, extension of this work to GGA+U would be straightforward and may be required particularly for studies of monolayers; previous studies have shown that GGA+U is necessary in order to obtain the correct Mott insulator behavior of monolayer 1T-TaS_2_,^51^ for which a significant on-site Hubbard energy *U* of 2.27 eV was obtained via the linear response method.^52^

In Figure 3d, we show the calculated 4f binding energies for the normal (i.e, non-CDW) phase of 1T-TaSe_2_ in which all Ta atoms are equivalent and only the two spin–orbit split components 4f_5/2_ and 4f_7/2_ are expected; in Figure 3c, we show results for the CCDW phase in which the tip, ring, and center Ta atoms now have clearly different core level binding energies. As noted above, the calculation of Figure 3c neglected spin–orbit splitting for convenience, and we should not compare absolute binding energies. The splitting between the tip and the ring sites is experimentally significant, but the peak of the center atom overlaps that of the ring atoms. In Figure 3c, the peaks are *not* weighted by the number of atoms of each type in the unit cell (t:r:c = 6:6:1); when this is done, it is clear that the center atoms are even less likely to be resolved experimentally. We can compare these predictions to recent experimental results of ours on the closely related material 1T-TaS_1.2_Se_0.8_, Figure 3e, where the dotted line represents the experimental data and the solid lines are fits using four components having the Doniach–Sunjic line shape.^53^ Consistent fitting including a contribution from center Ta atoms was not possible, in agreement with all previous studies of both 1T-TaS_2_ and 1T-TaSe_2_, but gave a splitting of the ring and tip contributions of ∼640 meV at 100 K and an intensity ratio between the 4f_7/2_ peaks of ∼1.2, consistent with t:(r + c) = 6:7. This is very similar to previous experimental results of, for example, ∼660 meV for 1T-TaSe_2_ at 70 K.^54^ For comparison, the present calculations give a ring–tip splitting of 633 ± 11 meV, with the spread depending systematically on the exact choice of input parameters.

The excellent agreement we obtain with experiment suggests that our simple approach is valid and will be of further use in investigating the rich field of Ta-based CDW and Mott insulator materials; the diversity of CDW behavior in this system has been noted and has certainly not yet been fully explored^49^ with complicated hysteretic behavior as a function of temperature, ultrafast dynamics,^55^ photodoping,^56^ intercalation,^57^ alloying and doping for superconductivity,^58,59^ and monolayer^60^ and heterostructure^61^ effects all being of current interest. The well-resolved nature of the 4f XPS peaks and their good signal-to-noise makes them a useful probe of the local Ta environment that is accessible in studies of all of the above types.

## Surface Core Level Shifts of MXenes

**Figure 4:**
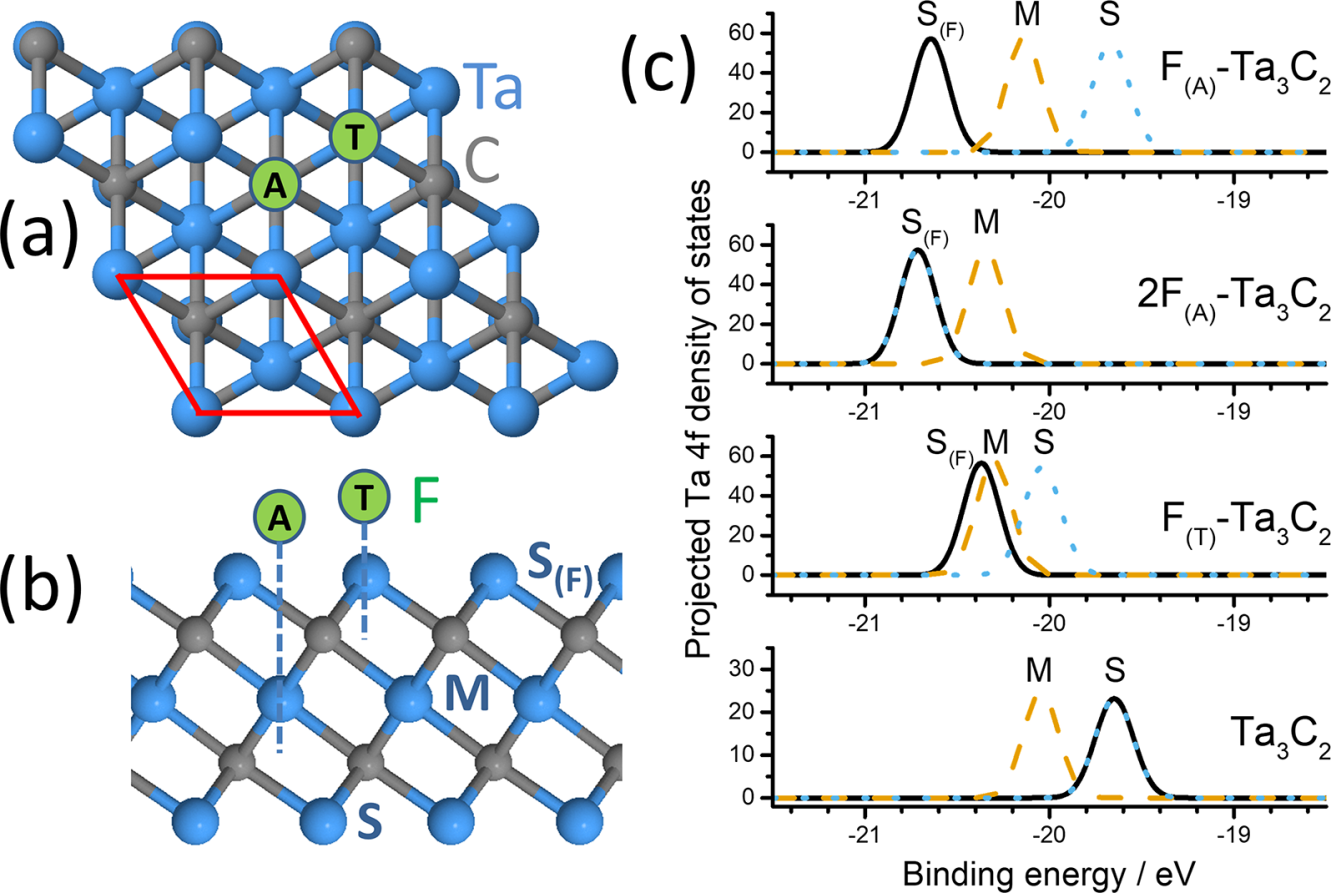
Structure and predicted XPS spectra of the example MXene Ta_3_C_2_. (a and b) Top and side views, respectively, of the MXene structure with three Ta layers (blue) and two C layers (gray); hexagonal unit cell is shown in red in a, and possible fluorine atom sites A and T are shown in green. (c) Projected Ta 4f density of states (without spin–orbit splitting) showing the SCLSs between middle (M) and surface (S) Ta atoms for four F configurations: from top to bottom per unit cell, one F on site A, two F’s on opposite A sites, one F on site T, and Ta_3_C_2_ without F. S_(F)_ indicates the peak arising from surface Ta atoms on the same side of the layer as the F atom. (Solid line) Ta atoms adjacent to the F atom; (dashed orange line) middle Ta atom; (dotted blue line) Ta atoms on opposite sides of the layer.

The MXenes are a class of 2D materials with a similar structure to the TMDs but with a very different chemistry and different manufacturing routes;^62,63^ unlike the TMDs, there are no naturally occurring MXenes. However, the MXenes may contribute a significant new range of properties to the suite of layered materials. They consist of alternate hexagonal planes of carbon atoms (labeled X since, in principle, nitrogen can also play this role) and metal atoms M, with the M atoms forming the layer surfaces, so having a composition M_*n*+1_X_*n*_; the *n* = 2 case is shown in Figure 4a and 4b. They are derived from the so-called MAX structures in which adjacent MXene layers are metallically bonded via, for instance, an intercalated layer of aluminum atoms (labeled A in the generic term MAX).^63^ There is no van der Waals bonding in the parent MAX structure, and so one can expect a chemically reactive and easily modified M surface after 2D layers are extracted by, for example, etching the aluminum away with hydrofluoric acid; one also expects to obtain surfaces terminated by F, O, and OH after this etching.

Much of the early DFT-based modeling of MXenes considered structures of pure M_it *n*+1_X_*n*_ type as shown in Figure 4a and 4b without surface termination,^64^ but recent experimental and computational work has addressed the question of termination by, for example, oxygen, fluorine, and hydroxyl groups.^65,66^ Here, we do not aim to give an exhaustive study of the MXenes, but we demonstrate via the example material Ta_3_C_2_ that we can again make predictions of the XPS spectra and, thus, that XPS is likely to be a very useful characterization tool. We carried out calculations on the simplest Ta-based *n* = 1 MXene, Ta_2_C, in order to check that the lattice parameter (*a* = 3.037 Å) obtained using our pseudopotentials was consistent with earlier work,^64,67^ but as this structure only contains one type of Ta site, it is not of direct relevance here.

However, in *n* = 2 Ta_3_C_2_ we predict a significant difference in the 4f binding energy for Ta at the central (M) and surface (S) positions, as shown in Figure 4c (bottom). In agreement with previous DFT modeling of this MXene,^64,68,69^ we find that an isolated monolayer of Ta_3_C_2_ is metallic (as is Ta_2_C) with an in-plane lattice parameter of *a* = 3.051 Å, in agreement with *a* = 3.086 Å from Lane et al.^69^ Our main finding here is that we expect experimental XPS to be able to discriminate between Ta atoms at M and S positions (here split by *E*_M_ – *E*_S_ = −326 meV, which is easily detectable with standard fitting procedures) and therefore that XPS should also be a useful indicator of surface modifications that will affect primarily the surface atoms. This prediction is as yet untested experimentally; there have been many XPS studies of other industrially important tantalum carbides from which it is known that the Ta 4f levels show a sensitivity to the Ta oxidation state,^70,71^ but this MXene remains to be investigated by XPS.

Although we have focused on the case of Ta, similar calculations of relaxed, unterminated M_3_C_2_ layers with M = Hf, Ta, W, and Re give *E*_M_ – *E*_S_ = +630, −326, −940, and −520 meV, respectively, showing that, in all cases, there is a considerable core level shift for the surface metal atoms. We note that partial surface termination will of course introduce nonequivalent M sites even in M_2_C, and so our simple approach may also be a valuable guide to the fitting of M_2_C XPS data.

In view of the likely surface termination of as-prepared MXenes, we also investigated whether surface termination can cause appreciable changes in SCLS, taking Ta_3_C_2_ as a model system. Following earlier work on titanium-based MXene Ti_3_C_2_,^72^ we consider the representative example of fluorine at two possible surface sites: A (above a middle Ta atom) and T (on top of a surface Ta atom), as shown in Figure 4a and 4b. We take the primitive unit cell shown in Figure 4a, so that we model complete occupation of all equivalent sites on one side of the layer and neglect spin–orbit effects for clarity. Figure 4c shows the results. Comparing each spectrum to the results without fluorine (bottom), we can recognize the SCLSs of the surface Ta atom on the side of the layer opposite to the F atom (S; dotted line) and the middle Ta atom (M; dashed line). Addition of fluorine at the A site introduces a new 4f band from the Ta atom to which it is bonded (top; solid line). Addition of fluorine at the A sites on *both* sides of the layer eliminates the S band and, since the bands plotted as overlapping will be summed experimentally, doubles the weight of the S_(F)_ band (second panel from top). Finally (second panel from bottom), addition of fluorine at the T site shifts the 4f level of the nearby Ta close to that of the M site. These four situations are quite distinct and will lead to different behavior when partial coverage is taken into account, showing that core level XPS should be a powerful tool to analyze surface termination effects.

## Conclusions

We have shown that for transition metals where the 4f states can be included as valence states of a pseudopotential, a single DFT calculation can give a simple initial state prediction of shifts in the 4f XPS spectra for nonequivalent transition metal sites within a given input structure. The shifts in 4f binding energy within one calculation reproduce the experimental results reasonably well (this was shown in the first section for the cases of low-index surfaces of Hf, Ta, W, and Re metal slabs and then for the CCDW phase of 1T-TaSe_2_). Our approach will not replace more accurate simulations but is easy to apply for the relevant transition metals and could provide useful physical input into the fitting of the XPS spectra of their less familiar compounds. As an example of one such new and very topical material, we chose the MXene M_3_C_2_ with undecorated or fluorine-terminated surfaces; we see clearly that the central and surface metal atoms are nonequivalent and differ significantly in 4f binding energy for all M = Ta, Hf, W, and Re and that fluorine termination also introduces measurable and site-dependent modifications of the SCLSs. The extension of our modeling to a M_*n*+1_X_*n*_ surface with O or OH termination would be straightforward for any *n* ≥ 1, as would the modeling of M_*n*+1_X_*n*_ with M = Ta, Hf, W, and Re and X = C and N.
